# How German health insurance providers use social online networks to promote healthy lifestyles: a content analysis of Facebook® accounts

**DOI:** 10.1186/s12911-021-01433-w

**Published:** 2021-02-18

**Authors:** Julika Loss, Charlotte von Uslar

**Affiliations:** 1 Department of Epidemiology and Health Monitoring, Robert Koch Institute, Berlin, Germany; 2 Medical Sociology, Faculty of Medicine, University of Regensburg, Regensburg, Germany

**Keywords:** Social media, Social networking sites, Internet, Health insurance provider, Sickness funds, Prevention, Health promotion, Health education, Physical activity, Nutrition

## Abstract

**Background:**

Social networking sites such as Facebook® can contribute to health promotion and behaviour change activities, but are currently underused for this purpose. In Germany, health insurance companies are relevant public health agencies that are responsible for health promotion, primary prevention, and health education. We intended to analyse the Facebook® accounts of health insurance providers to explore the range of prevention topics addressed, identify the communication formats used, and analyse user activity stimulated by prevention-related posts.

**Methods:**

We performed a quantitative content analysis of text and picture data on Facebook® accounts (9 months in retrospect) in a cross-sectional study design. 64/159 German health insurance providers hosted a Facebook® page, 25/64 posted ≥ 10 posts/months. Among those 25, we selected 17 health insurance companies (12 public, 5 private) for analysis. All posts were categorized according to domains in the classification system that was developed for this study, and the number of likes and comments was counted. The data were analysed using descriptive statistics.

**Results:**

We collected 3,763 Facebook® posts, 32% of which had a focus on prevention. The frequency of prevention-related posts varied among health insurance providers (1–25 per month). The behaviours addressed most frequently were healthy nutrition, physical activity, and stress/anxiety relief, often in combination with each other. All these topics yielded a moderate user engagement (30–120 likes, 2–10 comments per post). User engagement was highest when a competition or quiz were posted (11% of posts). The predominant communication pattern was health education, often supplemented by photos or links, or information about offline events (e.g. a public run). Some providers regularly engaged in two-side communication with users, inviting tips, stories or recipes, or responding to individual comments. Still, the interactive potential offered by Facebook® was only partly exploited.

**Conclusions:**

Those few health insurace companies that regularly post content about prevention or healthy lifestyles on their Facebook® accounts comply with suggestions given for social media communication. Still, many health insurance providers fail to actively interact with wider audiences. Whether health communication on Facebook® can actually increase health literacy and lead to behaviour changes still needs to be evaluated.

## Background

Throughout the developed world, the internet is now generally considered an indispensable communication tool [1]. It is currently based on the ‘Web 2.0′ standard, i.e. web applications allow end users to interact and collaborate as content creators, rather than to just receive one-directional information on static ‘Web 1.0′ websites dated pre-2004 [2, 3]. People gather online and interact by sharing information, media, knowledge, and opinions; internet platforms facilitating these interactions are known as ‘social networking sites’ or ‘social media’ [4]. Among the most widely used social media platforms are Facebook, YouTube, Instagram, Twitter, and Snapchat. Users of these sites usually upload personal details about themselves to their own ‘profile’ page, and then link their profile to the pages of their friends [5], allowing individuals to form and maintain social networks [6]. The last years have seen a tremendous growth in usage and popularity of these sites [2, 5].

Social media also has the potential to enhance public health communication [7, 8], as it can make health information more available and sharable, and tailor it to the needs of specific groups [9, 10]. Social media can also be used to provide social or emotional support [9], to empower people about health issues [7], and to understand public perceptions of health (policy) issues [8]. Public health organizations may face challenges when they intend to engage in social media interaction with the population; challenges include an organization’s insufficient understanding of social media, its lack of human resources dedicated to maintaining the communication with users, and competing with the confusing amount of available online information [2, 11].

Facebook and other social media platforms have been adopted by public health organizations for health promotion and behaviour change campaigns and activities [10, 12], e.g. concerning sexually transmitted diseases [13, 14], vaccinations [15], physical activity [16], or mental health [17]. These interventions were mostly specific projects driven and implemented by public health researchers. Apart from that, we do not know much about how public health agencies regularly use social media to interact with their users on an everyday basis [18]. Capurro et al. [9] state that ‘*the application of SNSs [social networking sites] to public health research and practice is still maturing*.‘ Several state and local health departments or Federal health agencies in the USA already use Facebook, or Twitter, to disseminate health information, e.g. about healthy lifestyles, communicable diseases, or infant and child health. Still, they hardly exploit the potential of social media in order to interact, engage and build relationships with users, or to address relevant local or regional health concerns [11, 18, 19].

No studies have looked into the social media communication of German (public) health organizations yet. In Germany, health insurance companies can be considered relevant public health agencies. Unlike other countries, German sickness funds (rather than state, regional or local authorities) are responsible for health promotion and primary prevention, and play an important role in health information and health education [20]. They issue leaflets and brochures and host websites informing about health- and illness-related topics [21, 22], offer courses about healthy lifestyles (e.g. physical activity, nutrition, or stress reduction [23]), and subsidize the participation in commercial exercise classes [24].

Health insurance is provided by circa 120 not-for-profit public health insurance providers (= statutory sickness funds), and about 40 private health insurance companies. The statutory health insurance rests on the principle of solidarity: each individual residing and/or working in Germany is obliged to conclude and maintain statutory health insurance, unless they earn more than the opt-out threshold (circa €60,000 per year). Those citizens with an income exceeding this threshold are allowed to (but do not have to) purchase private health insurance policies instead. About 87% of the population are insured through statutory sickness funds [24, 25], among which they can choose freely.

Both private and public health insurance providers have gained additional relevance in prevention and health promotion since 2015, when the Preventive Health Care Act came into effect. It stipulates that the different health insurance funds cooperate with the aim to enhance prevention and health promotion in various settings (schools, communities, workplaces). The health insurance providers are demanded to almost double their expenditure on prevention and health promotion [26, 27].

As health insurance providers play a significant role in prevention and health promotion in Germany, it is interesting to understand how they use social media for their health-related communication, especially if they make full use the potential of web 2.0. to interact with both their members and the population as a whole. As Facebook® is the social network that most German health insurance providers use [28], we decided to analyse the Facebook profiles of both public and private health funds with the aim of

- Exploring the range and proportion of prevention and lifestyle topics addressed by health care companies in social media.
- Identifying the different communication formats and preventive activities used.
- Analyzing the engagement stimulated by the prevention-related posts (i.e. number of ‘likes’), as indicators of the reach of the content.

## Methods

### Study design

We performed a quantitative content analysis of text and picture data on pages of the social online platform Facebook® in a non-experimental, cross sectional study design. We gathered Facebook® pages of German public and private health insurance providers and collected all posts for a time span of 9 months in retrospect.

### Classification scheme

For the development of a classification scheme, we first selected five big health insurance companies and analysed 330 posts (48–88 per provider) in an open, inductive way with regard to health topics, addressed lifestyles, preventive strategy, intervention types, and communication formats. The posts were coded by both authors independently, and results were discussed until consensus was reached. The final classification scheme is shown in Table 1.

**Table 1:** Classification system for categorizing the Facebook posts

1. Overall topic: health Does the post relate to a health issue, or any other aspect (e.g. premiums, job descriptions)?	A. Yes B. No
2. Targeted disease /condition	A. unspecific, general health B. specific disease / condition a. cardiovascular b. orthopaedic/musculoskeletal c. infectious diseases d. cancer e. mental diseases / mental health f. pregnancy g. type 2 diabetes
3. Lifestyle / behaviour The behaviour that is explained, illustrated, or promoted in the post	A. healthy nutrition B. exercise / physical activity C. stress and anxiety relief, relaxation D. dental / oral hygiene E. legal drugs (reducing / quitting / avoiding) F. protection from solar UV radiation and heat G. hygiene, prevention of infections, safer sex H. vaccination I. cancer screening J. safety at home / road safety / injury prevention
4. Type of information / communication The way the behaviour / disease is addressed in written form	A. appeal, invitation (to change behaviour) B. detailed information, educational content C. event notice (e.g. invitation to attend specific courses) D. prize game, contest, quiz
5. Preventive activity What is the type of preventive activity that the post asks the user to take part in – online or offline?	A. online information / education (to be read by user)/ prize game (to be executed online) B. offline educational event, health-related courses C. offline training, fitness courses D. phone hotline (giving background information) E. offline prize game / contest F. offline recreational activity (e.g. gymnastics session in the park, community walk)
6. Form of message / post In what format is the message conveyed?	A. text B. text & picture C. picture with inscription / title D. text & video E. video with inscription F. text with link G. link with inscription H. event
7. Interactivity	A. number of likes/dislikes B. number of comments C. number of shares D. number of markings E. number of questions

### Sample

For the analysis, we checked all 119 public and 40 private health insurance providers in Germany (according to the official online directory for health insurances, ‘www.krankenkassen.de’), 64 of whom hosted a Facebook® page. We only included health insurance companies with a minimum activity of 10 new posts per month (n = 25, two test months 09-10/2015). Among those, we selected 17 health insurance companies (12 public, 5 private), representing both health insurances operating on a national level (n = 12) and those operating on regional or federal state levels (n = 5). We excluded those agencies with the lowest posting frequency (< 1 prevention-related post per month), as there was no rich or representative data material to base the analysis on. We collected all posts on these 17 Facebook sites for the retrospective time span of 9 months (ca. 10/2015 to 06/2016).

### Analysis

The Facebook® pages were copied and stored using the tool Gadwin PrintScreen (64-Bit), including pages of commentaries. The screenshots were printed for analysis. Each post (picture or text message etc.) was given a code, which contained an abbreviation of the communicating health insurance company, the date of posting, and a short title for the content. After that, each post was classified with regard to all domains listed in Table 1. For example, a post consisting of a short text (two sentences) about the importance of immunization including a link to an educative video about vaccination would be assigned to the category “*infectious diseases*” in the domain “targeted disease / condition”, and to the category “*vaccination*” in the domain “lifestyle/behaviour”. The “type of information / communication” would be rated as “*detailed information, educational content*” due to the educative video. In terms of the domain “preventive activity”, the post would be categorized as “*online information / education*”, as there no offline course or vaccination possibility was pointed out. The “form of message / post” would be rated as “*video with inscription*”, as the text introducing the video is very short. The categories were charted in a Microsoft® Excel Data Sheet. All posts were classified and charted by one researcher (CvU); a random sample of circa 50 prevention-related posts was double-checked by the other author (JL) to control the accuracy of categorization. In addition, both researchers jointly reviewed the screenshot in ambivalent cases and re-coded the variable after reaching consensus. In addition, we counted the number of likes/dislikes, comments, shares and markings for each post as indicators of interactivity [8, 18]. Sometimes, a post addressed multiple categories of a domain (e.g. a post encouraging the user to eat healthily and move more). In this case, the post was counted once for each category (here: both healthy nutrition and physical activity); as a consequence the sum of post numbers addressing certain lifestyles could exceed the total number of posts. In addition, the kind of combination (healthy nutrition plus physical activity) was also recorded. The data were analyzed using descriptive analysis.

### Ethical aspects

All data that were screened and analysed for this study were collected from public Facebook accounts that were accessible for anyone. We did not collect any personal data. All health insurance providers included in the study received written information about the study process and gave informed consent. 14/17 health insurance companies additionally agreed that screenshots or pictures of their Facebook® sites could be shown in publications as figures in an anonymized form; quotes from those companies that gave no consent are not included in this article. According to a consultation with the ethics committee of the University of Regensburg (August 12, 2015), no formal application of an ethical approval was necessary, given the fact that we used only public pages and websites, and did not include personal data relating to single individuals (reference no. 14-160-0162).

## Results

### Health and prevention topics of the posts

We analysed 3763 Facebook® posts that were posted by the 17 health insurance companies over a timespan of 9 months. Of all posts, 44% (n = 1671) were dealing with a health topic; among those, 1191 had a focus on prevention (32% of the complete sample). The majority of health- or prevention-related posts were found on Facebook® accounts of the public health insurance companies (Table 2). The posts with no relation to health topics were, among others, premium changes, awards, or stories of the everyday work of the staff. Health-related posts that had no content about prevention were, among others, about organ donation, or medical or non-medical therapy of diseases (e.g. treatment of influenza symptoms, home remedies, diabetes management etc.).

**Table 2:** Proportion of posts referring to health or prevention topics

	Analysed posts (n)	Post with health-related content (n, %)	Post with prevention-related content (n, %)
Public health insurance providers (n = 12)	2913	1544 (53.0%)	1093 (37.5%)
Private health insurance providers (n = 5)	852	129 (15.1%)	98 (11.5%)
Total	3,765	1673 (44.4%)	1191 (31.6%)

**Fig. 1:**
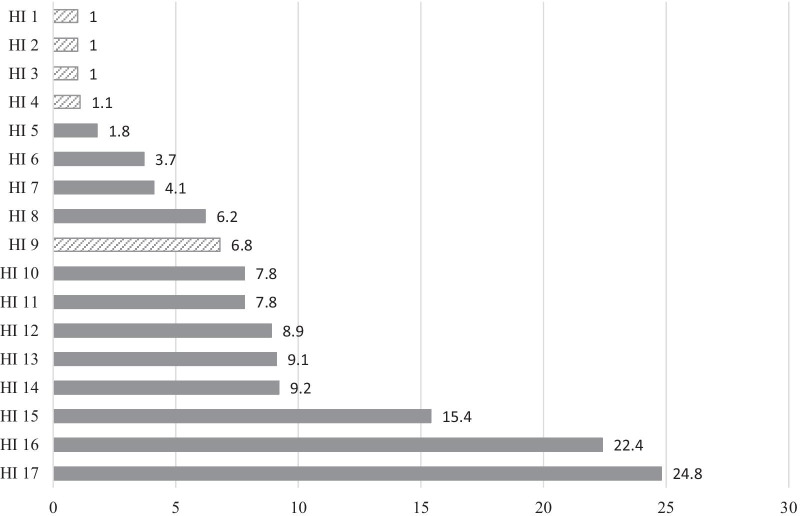
Prevention-related posts per month (n), by different health insurance providers (HI). The figure shows the average number of monthly Facebook® posts with content related to prevention, basing on the total numbers of posts collected over 9 months. Private health insurance providers are presented with a striped pattern (HI 1–4 and 9)

The following analyses refer only to the posts with prevention-related content (n = 1191). The number of prevention-related posts in the included timespan varied considerably (Fig. 1).

### Targeted conditions and lifestyles

A fifth of posts (20%, n = 241) explicitly dealt with (preventing) a certain disease or condition, mostly infections (n = 60), musculoskeletal diseases (n = 51), diabetes (n = 30), cardiovascular disease (n = 25), cancer (n = 25), mental illnesses (n = 24), or pregnancy (n = 26).

The majority of posts (80%) referred to health promotion in general (e.g. physical activity, healthy nutrition), without naming a specific diseases to be prevented.

Three health-related behaviours, i.e. ‘healthy nutrition’, ‘exercise/physical activity’ and ‘stress and anxiety relief/relaxation’ made up more than two thirds of all prevention-related posts (n = 912, 68%).

Posts on the topic of ‘exercise/physical activity’ mainly include information about events (e.g. city runs, sports groups), advice on correct sports equipment (e.g. bicycle) or point out activities in schools, companies or at trade fairs. In connection with risk reduction for specific diseases, there are calls for more exercise in everyday life. Posts on ‘healthy nutrition’ often consisted of recipe suggestions. In addition, nutritional and cooking courses were offered, or food with a special health-promoting effect (e.g. ‘for a good immune system’) was presented. ‘Stress and anxiety relief /relaxation’ was addressed by suggesting relaxing activities or meditation, or by giving advice on better sleep. Sometimes tests concerning personal (di)stress were made available.

**Fig. 2:**
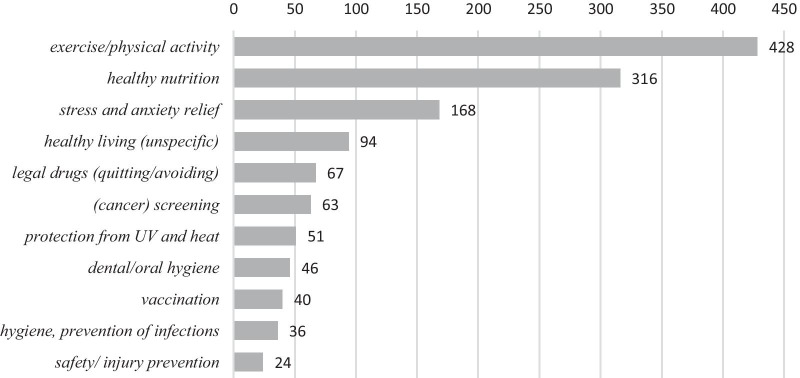
Frequency of behaviours/lifestyles addressed in post. The figure shows in how many posts a certain lifestyle or health-related behaviour was addressed, i.e. promoted or explained. 92 posts addressed two or more behaviours; as all addressed health behaviours were recorded and counted per post, the numbers add up to 1,333 addressed health behaviours for 1191 posts

Figure 2 shows the number of posts explaining or promoting the different lifestyles and behaviours.

We found 92 posts that addressed two or more behaviors, in 42 different combinations. The most frequent combination consisted of ‘healthy nutrition’ and ‘exercise/physical activity’ (n = 21), or ‘healthy nutrition’, ‘exercise/physical activity’ and ‘stress and anxiety relief’ (n = 17).

### Type of information/communication and preventive activity

Among the communication techniques used in the posts, the form of ‘detailed information, educational content’ was the most common (59.3%), with a large proportion of the health insurance companies included using all possible techniques (events, competitions, appeals), see Table 3.

**Table 3:** Frequency of different information types and communication techniques

	Posts (n, % of all prevention-related posts)	Case sample	Health insurance providers offering this technique (n)
Detailed information, educational content	713 (59.3%)	‘Once again we have a topical question of the week for you. Today we will be dealing with the important topic of early detection of skin cancer. Our position and what we can do for you, you can find out as always at [link]’. (*public health insurance provider*) ‘First a tiring meeting at work, then grocery shopping and the housework has to be done. For many parents this means pure stress. Here are some tips for you on how to escape the hustle and bustle of everyday life:... ‘ (*public health insurance provider*)	17/17
Event notice	251 (21.0%)	‘The day after tomorrow the B2Run company running championship will continue with the run in #Karlsruhe. Of course we'll be there again and maybe you can have your picture taken at our photo campaign. […] There are still last-minute starting places—all information about the run can be found here: [link]’ (*public health insurance provider*) ‘To all new mothers …: On October 12th we invite you to our expert evening ‘Breastfeeding—Food for Body and Soul’. XY, a breastfeeding and lactation consultant and training officer, will talk about important aspects of breastfeeding […]. YZ, owner of the Bra Fit Studio, will give important tips on the correct fit of the nursing bra. The free event begins (…)’ (*public health insurance provider*)	15/17
prize game, contest, quiz	134 (11.1%)	‘Send us your sportiest photo for winning a fitness tracker from XX! Just post a picture in the comments that shows you full of #Action! Because the motto is: #Steps, steps, steps! (*private health insurance provider*) ‘Win a „StressBusters “ service—deadline for participation ends today. You are studying and …busy with assignments and exams? If so, the (staff of the) StressBusters clean up for you. Because in a clean and tidy place, you can concentrate on your education. [link] (*public health insurance provider*)	12/17
Appeal, invitation (to change behaviour, to attend courses etc.)	105 (8.7%)	‘You won't have to give up on the Easter egg. But you should keep it at three eggs a week. Contrary to widespread opinion, studies have shown that the consumption of chicken eggs does not lead to an increase in cholesterol levels. On the contrary: since eggs contain many important nutrients, they make an important contribution to a varied and balanced diet. So enjoy your meal and we wish you a happy Easter!’(*public health insurance provider*)	15/17

The table shows the type of communication used in the studied Facebook® posts (n = 1191, multiple categorizations possible). Most posts were combined with a photo, which did not change the information category

The majority of prevention measures referred to in the posts were constricted to the online setting, i.e. consisted of informative texts, links to other pages, or educational videos (n = 925, 77%). A quarter of the posts (23%, n = 274) advertised activities, offers or events taking place ‘offline’. These offline offers comprised different categories: recreational events such as walking with a famous sportsperson, or family days in public swimming pools (n = 130, 11%), information events such as health-related courses (n = 66, 6%), training or fitness courses (n = 35, 3%), and phone hotlines (n = 29, 2.4%). A further offline activity consisted of competitions in which participants were asked to complete a task ‘offline’ and then, for example, upload a photo of it on the respective Facebook® page (n = 14, 1.2%).

### Form of the message/post

Of all preventive posts, 49% consisted of texts, most of which also contained visual material (photos: 10.3%, videos 1.9%) and/or links (37%); the informative focus was on the text element. Many posts (36.4%) had a link as key element, and 42.7% of posts consisted of photos, videos or links with subtitles or short introductions. Text-only posts were the exception.

### Activity

On average, the interactivity between posts of different health insurance providers varied between 3.2–572 likes/dislikes per post (mean: 82.3), and 0.1–72 comments per post (mean: 6.2), and were shared between 0.1 and 45 times (mean 9.6). In the whole sample, there were only 3 prevention-related posts which had no likes, shares or comments i.e. no activity at all. On the other hand, only 223/1191 posts (18.7%) had 100 or more total shares, likes and comments. The highest number of likes, shares and comments for a single post were 6129 likes, 858 shares and 629 comments, respectively. The top 5 posts ranked by activity per post were (1) a recipe for a green smoothie, (2) a program containing information about healthy sexuality, (3) a call for a prize game about attractive design of healthy food, (4) a program explaining how exercising could influence health, (5) a prize game in which users could win a bike by naming their preferred biking destination. Table 4 gives more details about the Facebook engagement metrics of different kinds of posts.

**Table 4:** Engagement metrics associated with different behavior topics and communication techniques

	Posts (n)	Posts with zero activity (n)	(Dis)likes/ post (n)	Shares/ post (n)	Comments/ post (n)	Responses on comments/ post (n)
**Lifestyle/behaviour addressed in post**						
Exercise/physical Activity	428	1	84.3	7.8	7,5	1,3
Healthy nutrition	316	0	97	9.6	9,4	2,2
Stress and anxiety relief	168	0	59.2	12.6	2,6	1,3
Healthy living (unspecific)	94	1	121	10.1	4,0	2,7
Legal drugs	67	0	73.2	11.2	3,6	1,5
Cancer screening	63	1	53.4	16.2	2,2	1,6
Protection from UV / heat	51	0	61.9	7.4	1,8	1,4
Dental / oral Hygiene	46	0	30	3.5	1,8	0,6
Vaccinations	40	0	41.3	3.45	1,2	3,4
Hygiene, prevention of infections	36	0	42.1	4.4	1,7	1,1
Safety / injury prevention	24	0	106.9	12	3,3	2,3
**Type of information / communication**						
Detailed information, educational content	713	2	80.3	10.3	4,2	1,8
event notice	251	1	29.4	5.3	0,6	0,2
Prize game, contest, quiz	134	0	171.8	17.2	37,2	4,0
Appeal, invitation (to change behaviour, to attend courses etc.)	105	0	129	5.7	3,5	1,2

## Discussion

### Principal findings

Only a small minority of German health insurance agencies made use of the online social networking site Facebook to interact regularly with their clients or wider audiences. About a third of all posts on those Facebook sites were about prevention, e.g. health education about certain lifestyles, or announcement of courses or preventive activities. The health insurance companies differed considerably in the frequency with which they dealt with preventive topics in their Facebook posts—between 1 and 25 prevention-related posts per month. Strikingly, the private health insurance companies ranked lower in this regard than the public health insurance providers. The lifestyles and behaviours that were addressed most frequently were healthy nutrition, physical activity, and stress and anxiety relief, often in combination with each other. Fewer posts dealt with consumption of legal drugs, prevention of cancer, protection from UV radiation, or vaccination. All these topics yielded a moderate user engagement of about 30–120 likes and about 2–10 comments per post on average. User engagement was highest when the health insurance companies posted a competition or a quiz, which made up, however, only about a tenth of prevention-related posts. The predominant communication pattern was conveying information and educating about certain prevention topics and behaviours—often supplemented by photos or links -, or informing about offline events (e.g. a public run). The health insurance companies only partly exploited the interactive potential offered by social media such as Facebook, although some providers regularly engaged in two-side communication with users by responding to individual comments.

### Comparison with other studies

Thackerey et al. [8] analysed the social media usage of 50 state health departments in the USA in 2011 and found that 34% had a Facebook® account, quite similar to 40% in our sample of German health insurance companies. That US study found a much higher rate of health-related posts among all posts, as compared to our findings (88% versus 44%). The most common topics were influenza, environmental health, heart disease, nutrition, tobacco, emergency preparation, and cancer. As Thackerey et al. did not distinguish between general health topics (e.g. diagnosis, treatment) and prevention, it is hard to compare these topics to those identified most frequently among the posts in our sample (physical activity, nutrition, and stress relaxation), which only included prevention-related posts. In the study by Thackerey et al., 16% of posts were event announcements, which is comparable to 21% in our sample. Another study from the USA [18] analysed the posts of Facebook® accounts belonging to 24 health agencies, such as the Center for Disease Control & Prevention, the Food & Drug Administration or the National Eye Institute. This study focused on the differences of interactivity between the different health agencies, and found that e.g. “likes” varied between 1 and 34 per post among the included agencies (as compared to 3.2–527 among the health insurance providers in our sample), and the number of comments per post ranged from 0.2–11 (versus 0.1–72). Differences can be explained by the selection of posts (we only analysed prevention-related posts, whereas Bhattacharya et al. included all posts). That study [18] also categorized the content and semantics of the Facebook posts, but the results cannot be compared as the chosen categories were on a completely different level (e.g. “concepts & ideas”, “disorders”, “anatomy”, “genes and molecular sequences”, or “geographic areas”).

### Strengths and weaknesses

To our knowledge, this is the first content analysis of social networking sites hosted by German health agencies, in this case health insurance companies. Limitations include the cross-sectional design of the study; online social networking is changing quickly, and this change could not be captured adequately, even though we included posts of a retrospective timespan of 9 months for each health insurance company. It may also be a limitation that we restricted our study to the social network Facebook; some health insurance companies may also use other social media platforms to interact with their members or the public, e.g. Twitter. Kühne et al. [28] had identified Facebook® to be the social network used most frequently by German health insurance providers; in addition, we performed a trial research at some exemplary selected health insurance companies, and found that social media other than Facebook were rarely used. A further limitation is that there is no universally accepted standard procedure for analysing social media content with regard to prevention. When selecting indicators and categories, we drew on the literature wherever possible, e.g. in recording the site’s interactivity with users. We could not find a scheme which detailed categories for prevention topics and specific communication techniques used in association with prevention (e.g. inviting for offline activities and courses, prize games in which fitness gear fostering physical activity could be won); therefore, we had to come up with novel indicators and classifications, which were developed in a first inductive part of the study. It can be considered a strength that we did not simply search for pre-defined keywords; instead, we thoroughly read each of the ca. 3,800 posts and assigned them individually to the respective categories.

### Implications for policy and practice

Our study confirms the observation that many public health agencies still struggle to reach larger—and younger—audiences using social media [3, 29]. Of those 40% of German health insurance companies that hosted a Facebook account, only about a third posted 10 posts or more per month, and only a minority of these posts were about health or prevention. This is even more disappointing, as the analysis was performed after the German Preventive Health Care Act had come into effect. With this Act, the statutory and private health insurance providers have been given the task of strengthening prevention in Germany, and were obliged to allocate substantial financial resources to prevention [26]. Given this background, health insurance companies have to consider how the world now operates with online, mobile technologies and social networking applications, and how this relates to health promotion and health education [3]. This does not only entail that health insurance companies should start and/or extend to use social media such as Facebook®, Twitter® etc. in order to reach out for their members and wider audiences; it does also mean that they use these applications in a meaningful, engaging, and entertaining way.

As Thackerey et al. note, ‘social media is more than another communication channel’ [8]. Apart from just educating about health topics, social media present many opportunities for interacting, communicating, and building relationships with various audiences. The health insurance providers with an active Facebook site have partly taken these features into account. In our sample, the majority of posts consisted of informative texts or educational content, or advertised offline activities. On the other hand, we also found that many posts capitalized on internet resources, by providing links or posting videos. Multimedia has proven to be engaging for users because of its direct impact on various senses, including the photo format [30], which is very frequently employed by the studied health insurance companies. A substantial number of posts explicitly invited the users to share their experiences and recommendations (e.g. on stress relaxation, staying fit, or healthy cooking), and 10% of posts were prize games or competitions. Those posts created a remarkable user activity of an average of more than 170 likes, 17 shares and 37 comments per post, which is relatively high also compared to activity metrics that have been published for different US public health agencies’ Facebook posts. Health insurance companies (or public health agencies, respectively) can evaluate the activity generated by different kinds of posts and regularly employ strategies that succeed in engaging users in an entertaining and stimulating way.

Klassen et al. suggest that health promotion organizations learn from effective social media techniques employed by the lifestyle and food industry. For example, strategies inducing positive emotions and using an optimistic tone could yield more user activity on Facebook® [29]. We found that indeed many analysed posts had a positive message, a cheerful tone and a somewhat chummy language relating directly to the user. Typical examples were recipes for ‘modern’ healthy eating (e.g. smoothies, superfoods), advice on sporting gear, or tips for winding down and relaxing. While these communication styles are in line with the suggestions for positive emotions, one may discuss that they come at the cost of other more serious topics and appeals, which may be of equally high, or even higher, public health relevance, e.g. quitting smoking, drinking less alcohol, or cancer screening. The high frequency of the topics physical activity, nutrition, and stress relaxation could therefore be the result not only of an organization’s public health concerns, but also its intention to post positive, friendly, contemporary messages which could easily be supplemented with attractive, appealing pictures (of fruits, of a person relaxing in a hammock). One may also speculate that the health insurance providers may also use their Facebook posts to advertise themselves and present themselves in a favourable way. A study of Facebook communication of young people, for example, showed that they do not simply report upon their health-related behaviour, but also utilize these reports as a form of positive self-presentation. Health topics such as physical activity or shared (un)healthy meals served for the youth’s impression management on Facebook® [31]. These finding may be somewhat related to the predominance of ‘positive’ health topics on the Facebook® sites of health insurance companies, which are in fact in competition with each other for (young, healthy) members. Health topics that sound more serious or threatening, such as cancer or the dangers of legal addictive drugs, should not be forgotten; if social media do not prove to be a suitable medium for this, other communication channels must be chosen to compensate.

According to a scoping review by Joseph-Shehu et al. [32], physical activity, healthy diet and sexual health are the lifestyles that are addressed most frequently by information technologies such as social networking sites, websites, or short messaging service, and the results of the interventions were encouraging. Likewise, a meta-analysis by Laranjo et al. [33] identified a positive effect of interventions on social networking sites (such as Facebook®) on health behaviour-related outcomes, especially physical activity and weight loss, although there was considerable heterogeneity. The social networking components of the interventions were primarily used to provide health education and social support. Pairing the informative elements with monitoring tools may help improve physical activity and weight loss [32].

We have not checked if the health information and advise given in the Facebook® posts were evidence-based; neither do we know whether they are effective in changing the users’ awareness, knowledge or behaviour with regard to certain lifestyles or preventable diseases. In an older study, Mühlhauser et al. [21] found that health information on the websites of two German statutory health insurance agencies failed to meet relevant criteria of evidence-based consumer information; but it is not clear whether these findings also apply to the content of current social media applications. Further studies could therefore analyse in detail e.g. the nutritional value of the recipes and their respective contribution to a balanced diet. It would also be interesting to survey the users with regard to acceptance and effectiveness of the health advice and preventive information. This would be in line with the ‘public health agenda for social media research’ put forward by Pagoto et al. [34]; it suggests, among others, (a) to evaluate the impact of health-related social media initiatives, and (b) to identify the most effective strategies for generating meaningful user engagement which leads to healthy changes in knowledge, attitudes, and behaviour in online communities.

## Conclusion

Those few health insurance companies that regularly use their Facebook® accounts for health promotion and prevention already comply with many suggestions given for communication on social media, e.g. frequent use of a positive tone, real-world tie-ins, and regular invitations for users' co-production of knowledge. Still, many health insurance companies fail to capitalize on the vast potential of social media to actively interact with wider audiences. Further studies are warranted to understand whether the posts dealing with prevention and health promotion can actually increase health literacy and lead to healthy changes in lifestyles and behaviour of the users. According to the literature, addressing the topics physical activity, healthy diet as well as mental and sexual health may be most promising. It may also be interesting to analyse whether the selection of topics particular suitable for social media applications may lead to an imbalance in the representation of health risks, by favouring the appealing ‘lifestyle’ topics over the more serious, disturbing facts.

## Data Availability

The datasets used and/or analysed during the current study are available from the corresponding author on reasonable request.

## Abbreviation

US, USA: United States of America
